# Ontogenetic trajectories of body coloration reveal its function as a multicomponent nonsenescent signal

**DOI:** 10.1002/ece3.4369

**Published:** 2018-12-07

**Authors:** Willem Bonnaffé, Mélissa Martin, Marianne Mugabo, Sandrine Meylan, Jean‐François Le Galliard

**Affiliations:** ^1^ UPMC Univ Paris 06 CNRS IRD INRA Institut D’écologie et des Sciences de l'environnement (iEES) Sorbonne Universités Paris France; ^2^ Département de Biologie Ecole Normale Supérieure PSL Research University Paris France; ^3^ School of Biology Faculty of Biological Sciences University of Leeds Leeds UK; ^4^ Paris‐Sorbonne Univ Paris 04 ESPE de l'académie de Paris Sorbonne Universités Paris France; ^5^ Centre de Recherche en Écologie Expérimentale et Prédictive (CEREEP‐Ecotron IleDeFrance) Ecole Normale Supérieure CNRS UMS 3194 PSL Research University Saint‐Pierre‐lès‐Nemours France

**Keywords:** age‐dependent coloration, carotenoids, senescence, structural coloration

## Abstract

The understanding of developmental patterns of body coloration is challenging because of the multicomponent nature of color signals and the multiple selective pressures acting upon them, which further depend on the sex of the bearer and area of display. Pigmentary colors are thought to be strongly involved in sexual selection, while structural colors are thought to generally associate with conspecifics interactions and improve the discrimination of pigmentary colors. Yet, it remains unclear whether age dependency in each color component is consistent with their potential function. Here, we address lifelong ontogenetic variation in three color components (i.e. UV, pigmentary, and skin background colors) in a birth cohort of common lizards *Zootoca vivipara* across three ventral body regions (i.e. throat, chest, and belly). All three color components developed sexual dichromatism, with males displaying stronger pigmentary and UV colors but weaker skin background coloration than females. The development of color components led to a stronger sexual dichromatism on the concealed ventral region than on the throat. No consistent signs of late‐life decay in color components were found except for a deceleration of UV reflectance increase with age on the throat of males. These results suggest that body color components in common lizards are primarily nonsenescent sexual signals, but that the balance between natural and sexual selection may be altered by the conspicuousness of the area of display. These results further support the view that skin coloration is a composite trait constituted of multiple color components conveying multiple signals depending on age, sex, and body location.

## 1 INTRODUCTION

Intraspecific variation in the expression of biological ornaments is ubiquitous and has received considerable attention because body coloration often signals individual quality during fighting and mating interactions (e.g. Galván & Møller, 2009). Yet, biological ornaments are complex, multicomponent signals that convey different messages to congeners according to the reflectance properties and location of the color patch on the body, and functional correlations with other nonvisual signals (Cuthill et al., 2017; Grether, Kolluru, & Nersissian, 2004). Individual color components can be divided in two categories including either structurally based coloration, such as ultraviolet (UV) reflectance that originates from ultra‐structure properties of the color patch, or pigmentary‐based coloration that originates from pigment absorption and often plays an important role in sexual selection (e.g. Svensson & Wong, 2011), such as yellow–orange carotenoids (Martin, Meylan, Gomez, & Le Galliard, 2013). Furthermore, the same color component may signal different characteristics depending on the body location and sex of the bearer. For example, different pigmentary color patches may signal sexual receptivity, social dominance, or mate quality (LeBas & Marshall, 2000; Ord, Klomp, Garcia‐Porta, & Hagman, 2015). Thus, studies of intraindividual variation can critically help understanding how differences in body coloration are generated and which selective mechanisms might be involved (e.g. Badyaev, Hill, Dunn, & Glen, 2001; Evans & Sheldon, 2013; Galván & Møller, 2009).

However, previous studies of intraindividual variation in body coloration have generally failed to account for the complexity of visual signals by not examining jointly distinct color components in different body regions. In addition, although most conspicuous color traits are to some degree positively age dependent, the shape of their age‐related variation, especially senescence patterns, remains unclear (Bradley, Hubbard, Jenkins, & Safran, 2014; Evans, Gustafsson, & Sheldon, 2011; Evans & Sheldon, 2013; Galván & Møller, 2009; Torres & Velando, 2007). The study of age dependency in color signaling, and more particularly color ontogeny and senescence, can further our understanding of the function of individual color components. First, ontogeny of body color, which is the development of individual color components in early stages of life, can be related to the life history of individuals (Booth, 1990; Oh & Badyaev, 2010; Olsson, Stuart‐Fox, & Ballen, 2013). More particularly, color components with a rapid onset may play a role in juvenile life stages, for instance by regulating intraclass competition among juveniles, while color components with an onset around the age of maturity are more likely to be involved in sexual selection (Grunst, Rotenberry, & Grunst, 2014). Second, the study of senescence of color components, that is the decay of individual color components in late life, provides hints for the selective pressures acting on them (Galván & Møller, 2009). In general, the maintenance and display of a conspicuous coloration is costly, and late‐life decrease in the expression of color components is expected from natural selection (Evans et al., 2011). Conversely, sexual selection should favor an increased expression with age of color components critical for mating success (Evans et al., 2011). Similarly, organisms where reproductive outputs typically increase with age/size such as in many indeterminately growing species of ectothermic vertebrates may not be subjected to senescence (Vaupel, Baudisch, Dölling, Roach, & Gampe, 2004). Altogether, the ontogeny of color signals that are not involved in sexual selection should be identical in males and females and show sign of senescent decay only if they are costly to produce and maintain. On the other hand, the ontogeny of color signals involved in sexual selection should be different in the two sexes, and should not be subjected to senescence as a result of a terminal investment strategy (Cote, Le Galliard, Rossi, & Fitze, 2008). Yet, it remains unclear whether these patterns of age dependency of color components are consistent with their supposed functions and the conspicuousness of the display (e.g. different body areas).

To further our understanding of the link between ontogeny and function of body color, we analyzed age‐dependent variation in body coloration from a longitudinal study of a cohort of common lizards (*Zootoca vivipara*) measured repeatedly throughout life until the age of 4 years old, where reproductive senescence starts (Massot et al., 2011). This species is characterized by a conspicuous UV and yellow–red carotenoid based ventral coloration extending from the throat to the belly and involved in social dominance and mate choice (Martin et al., 2013). Skin coloration is produced by dermal chromatophore units including a first layer of xanthophores where carotenoids are located, a second layer of iridophores where light is scattered and reflected by crystals in short and long wavelengths, and a third innermost layer of melanophores (Grether et al., 2004; San‐Jose, Granado‐Lorencio, Sinervo, & Fitze, 2013). We thus quantified three major color components, including carotenoid pigmentary, UV structural, and skin background (i.e. produced by dermal chromatophores) structural colors (San‐Jose et al., 2013), in three body regions (throat, chest, and belly) that play different roles in social interactions (Martin et al., 2013). In the common lizard, adult males display stronger UV and pigmentary colorations than females, UV color intervenes in intrasexual competition among males for females, and pigmentary color potentially intervenes in intersexual selection such as mate choice in females (Martin et al., 2013; Martin, Meylan, et al., 2015; Vercken, Massot, Sinervo, & Clobert, 2007). Hence, an intense UV and pigmentary coloration is thought to be primarily a male sexual signal. These studies also reported that this sexual dichromatism was more marked on the chest‐belly region than on the throat (Martin et al., 2013). This, in addition to the fact that sexual harassment of females by males occurs in common lizards suggests that the cost of the display of intersexual signals should be magnified on the throat (Martin et al., 2013; Vercken et al., 2007). Yet, these color components have also been shown to play a role in social interactions, such as dominance, and are thus influenced by natural selection (Vercken et al., 2007). A priori, skin background reflectance should be an inexpensive color component to produce and maintain and might not be involved in reproduction (San‐Jose et al., 2013).

Based on this work, we can formulate predictions on the age‐dependent pattern of body color components of common lizards in each sex and each body location. First, UV and pigmentary colors should develop more strongly in males and should not be subjected to senescent late‐life decay, as intense UV and pigmentary colors are primarily thought to be a male sexual signal. Second, the development of sexual dichromatism in these two color components is expected to be stronger on the belly than on the throat due to the high cost of conspicuous display of sexual ornaments in this species. Finally, skin background reflectance should develop identically in both sexes and should not be subjected to senescent late‐life decay.

## 2 MATERIAL AND METHODS

### 2.1 Population and sampling scheme

We measured ontogenetic variation of ventral coloration of common lizards in semi‐natural populations housed in outdoor enclosures at the CEREEP‐Ecotron IleDeFrance nearby Paris (48°17′N, 2°41′E, France), where conditions are similar to natural habitats apart from protection against avian and terrestrial predators. Individuals from the same birth cohort were monitored from 2006 to 2010 with nine consecutive capture sessions during the activity season, where all surviving individuals were captured by hand. To monitor within‐individual changes, lizards were all marked at birth by toe clipping. At each capture, we measured body size (snout‐to‐vent length, SVL) to the nearest mm and coloration (see below). True age (in days) was calculated as the difference between the date of the measurement and the date of birth. To account for periods of inactivity, namely the wintering season where lizards are not growing and coloration is not changing, we excluded 5 months (from October to February) from the year‐to‐day conversion, resulting in 210 days long active years. Sexual maturation occurs around the age of 200 activity days in this population (J. F. Le Galliard).

### 2.2 Spectrophotometric measurements and data processing

**Figure 1:**
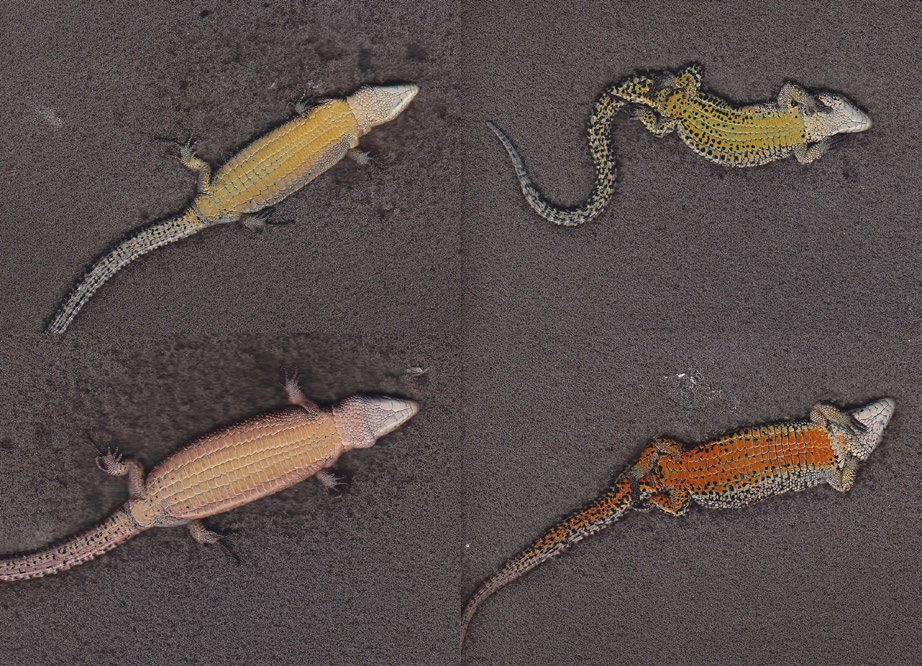
Digital photographs of the body color of individual common lizards. The individuals are an immature female (top left), an adult female (bottom left), an immature male (top right), and an adult male (bottom right)

Adult lizards display bright yellow–red colors on their belly and white–orange UV reflective colors on their throat (Figure 1), which are relevant to social interactions with conspecifics during territorial displays and to mate choice (Fitze et al., 2009; Martin, Meylan, et al., 2015). Ventral coloration was assessed by spectrophotometric measurements of the reflective properties of the skin in three body regions (throat, chest, and belly). Measurements were performed using a spectrophotometer (USB2000; Ocean Optics Inc.) calibrated between 200 and 850 nm, a Xenon light source (PX‐2) covering 220–750 nm range, and a 400‐μm fiber optic probe (R400‐7‐UV/VIS; Ocean Optics Inc.). Only the 300–700 nm range was considered for analysis because it corresponds to the perceptual range of vision in common lizards (Martin, Le Galliard, Meylan, & Loew, 2015). Measurements were obtained by placing and beveling the probe at a 45° angle on the lizards’ skin, avoiding black spots, resulting in a reading spot of approximately 1 mm^2^. Reflectance was measured relative to a dark and a white diffuse reference background (WS‐1; Ocean Optics Inc.). Two measurements were made on each body area of each lizard (for further details see Martin et al., 2013).

Spectral data processing was carried out using the *pavo* package (Maia, Eliason, Bitton, Doucet, & Shawkey, 2013) in R (v3.1.1). Spectra were first checked visually and aberrant spectra (308 spectra out of 24,691) as well as spectra of molting individuals (3,401 spectra) were removed. All spectra were then smoothed using the *procspec* function with a span set to 0.08, and all negative values of reflectance were set to 0. Reliable measures of aging require repeated measurements on the same individuals until senescence starts, but this usually reduces sample size resulting in low statistical power and biased samples due to selective filtering (Nussey, Coulson, & Gaillard, 2008). In order to reliably estimate growth trajectories until older ages without reducing too much the sample size, we thus extracted a subset of individuals (155 of 1,720) measured at least three times, at least once before reaching 100 days in age and once after reaching 200 days in age. We removed data taken before 2 months (<60 days) of age because ventral coloration was still brown or black for most individuals (pers. obs.). This final sample, which we used for the statistical analysis, thus included data from lizards aged 4 years (lizard that lived longer than 1, 2, 2.5, and 4 years approximately accounted for 75%, 50%, 25%, and 5% of the sample size, respectively) when demographic senescence is already significant in wild populations where the pace of life is slower than in our study site (Massot et al., 2011). As senescence is likely to occur earlier in populations with faster pace of life, such as ours (Mugabo, Marquis, Perret, & Le Galliard, 2011), we thus expected clear signals of aging in body coloration traits in our study sample.

We extracted three colorimetric variables to characterize distinct components of coloration: (a) the UV chroma (percentage of total reflectance between 300 and 400 nm), which quantifies the relative proportion of UV coloration (Martin et al., 2013; San‐Jose et al., 2013) and may be affected by structural and pigmentary components of the skin (Grether et al., 2004); (b) the violet–blue reflectance (reflectance summed between 400 and 515 nm, *R*_violetblue_), which is negatively correlated with the skin carotenoid content (San‐Jose et al., 2013); and (c) the skin background reflectance (reflectance summed between 575 and 700 nm, *R*_background_), which is representative of the structural coloration produced by the skin background layer of iridophores and melanophores (San‐Jose et al., 2013). We estimated the repeatability of spectrophotometric measurements by calculating the correlation coefficient between the first and second measurement of the same body region (*n *= 10,447); overall, we found high repeatability for all traits (*ρ*_UV chroma_ **= 0.95, *ρ*_*R*violetblue_ **= 0.93, *ρ*_*R*background_ **= 0.87).

### 2.3 Statistical analyses

First, we calculated the mean of the two measures per body location. Our initial investigations (ANCOVA with body location as a three level factor) showed that the only significant differences between chest and belly colorations consisted in a higher chest skin background reflectance in females (*p* < 0.01) and a stronger drop in UV reflectance with age on the chest of females (*p* = 0.03). In addition, previous studies on this species found no significant difference in average body coloration between chest and belly (Martin, Le Galliard, et al., 2015; Martin et al., 2013). Thus, here, observations from the belly and chest were pooled and averaged, and hereinafter referred to as the chest–belly region (CBR).

We first transformed data to meet the requirements of model assumptions, taking the square root of the violet–blue reflectance and the skin background reflectance to the 2/3 power. All continuous variables were then standardized with respect to their sample mean and standard deviation. The effect of body location, sex, and age, as well as their interactions, on each colorimetric variable and color contrast was estimated using a multiple regression in linear mixed effect models with the *lmer* procedure in R (Bates, Mächler, Bolker, & Walker, 2015). Each full model featured region‐dependent quadratic effects of age to account for potential nonlinearity and region dependency. In the result section, we refer to estimated linear effects as *β* and quadratic effects by the term *γ*. In addition, each model also included the identity of individuals as a random effect as the color of each individual in the final sample (*n* = 155) was measured at least three times throughout their lifespan and that for each color component and body region. Alternative models were also tested with body size as a covariate instead of age to characterize the relationship between each colorimetric variable and body growth. A stepwise backward model simplification was carried out for each full model until a minimum adequate model (MAM) was reached. At each step, the term minimizing the change in AIC upon deletion, and if the minimum change was <2, was removed from the full model. This step was repeated until the deletion of any of the terms would result in a 2‐unit increase in AIC, at which point the model was accepted as MAM. To be extra cautious, the deletion of a term was rejected if it led to a significant increase in deviance, tested using likelihood‐ratio tests (see Supporting Information Tables S1 and S3). Finally, we calculated Spearman rank correlations between the individual scores of the three colorimetric variables, which were calculated from the conditional modes of the random effects of the three minimum adequate linear mixed effect models.

## 3 RESULTS

### 3.1 Differences between body regions

**Figure 2:**
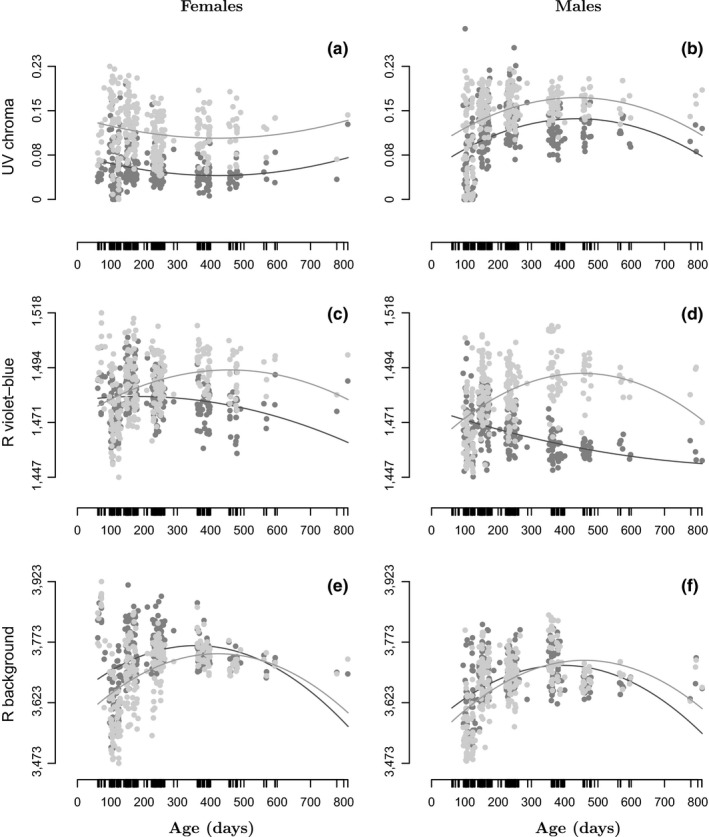
Change in UV chroma (a,b), violet–blue reflectance (c,d), skin background reflectance (e,f), along age predicted by models for females (a,c,e) and males (b,d,f). The solid lines correspond to changes predicted from best models in Supporting Information Table S2 for each body region (light gray: throat, dark gray: CBR) and sex

The mean UV chroma was lower on the chest–belly region (CBR) than on the throat, especially in females (Figure 2a,b, Supporting Information Table S2), and body regions had similar ontogenetic patterns for UV chroma. The two other colorimetric variables had both age‐ and sex‐dependent differences between body regions. *R*_violetblue_ was lower on the CBR than on the throat with increasing age, and the difference between CBR and throat was higher on average in males than in females (Figure 2c,d). *R*_background_ was slightly higher on the CBR than on the throat early in life, especially in females, and then reached similar values at older ages (Figure 2e,f).

### 3.2 Age‐related patterns and relationships with body size

The ontogenetic pattern for UV chroma was different between sexes (Figure 2a,b, Supporting Information Table S2). The UV chroma decreased and then stabilized with age in females, whereas it increased and then decreased with age in males. Ontogenetic changes in *R*_violetblue_ followed more distinct patterns on the CBR than on the throat and were further influenced by sex (Figure 2c,d). *R*_violetblue_ decreased slightly with age on the CBR of females (*β* = −0.06 ± 0.05, *p* = 0.23; *γ* = −0.07 ± 0.03, *p* = 0.04), while it increased on the throat of both sexes. The decrease of *R*_violetblue_ on the CBR with age was stronger in males than in females (Supporting Information Table S2). *R*_background_ increased and then decreased with age on the CBR, while it increased with age on the throat (Figure 2e,f). The increase was slightly lower in females than in males (Supporting Information Table S2).

Using body size as a covariate instead of age changed the ontogenetic patterns because body size growth is strong early in life (up to 2 years old) and then decelerates strongly with age (Supporting Information Table S4). Relationships with body size were almost linear for UV chroma (decrease in females and increase in males), throat *R*_violetblue_ (increase in both sexes), and *R*_background_ (increase in both sexes, see Supporting Information Figure S1). *R*_violetblue_ on CBR increased and then decreased with body size in both sexes, whereas it decreased rather linearly with age (see above).

### 3.3 Correlations between colorimetric variables

Altogether, there were significant interindividual differences for each colorimetric variable and for color contrast (c.a. 25%–35% of the variance components, Supporting Information Table S2). Individual scores for UV chroma correlated positively with *R*_violetblue_ (Spearman *ρ* = 0.46, *p* < 0.0001) but not with the *R*_background_ (*ρ* = 0.09, *p* = 0.25). Individual scores for *R*_violetblue_ correlated positively with *R*_background_ (*ρ* = 0.58, *p* < 0.0001).

## 4 DISCUSSION

The aim of our study was to characterize ontogenetic patterns of each component of the body coloration of common lizard and address differences between sexes and body locations. We expected ontogenetic patterns to be in line with the supposed function of each color component. Namely that UV and pigmentary colors would develop and be maintained predominantly in males, as they are thought to be male sexual signals, and that skin background color would develop and be maintained in late life identically in males and females, as thought to be inexpensive nonsexual signals. Furthermore, we expected that sex‐related differences in ontogenetic patterns would be smaller on the throat, as the display of sexual dichromatism is thought to be highly costly in this species. Our analyses uncovered distinct ontogenetic patterns between color components, sexes, and body regions, some of which challenge a priori assumptions regarding the selective pressures underlying the associated color signal. Overall, our results suggest that the function of a color component, along with associated selective pressures, most likely varies across sexes and area of display.

### 4.1 Ontogeny and function of body color

As was found in a previous cross‐sectional study (Martin et al., 2013), our longitudinal study indicates that UV chroma decreased with age in females but increased strongly with age in males and that in a similar way between the two body regions. This implies that strong UV coloration may be specific to older males, which further suggests that it may more readily signal male characteristics, such as sexual maturity or quality, rather than female characteristics. This would support earlier behavioral studies that demonstrated the role of throat UV coloration as a signal of dominance (i.e. territory holding capacity) and sexual attractiveness in male lizards (Martin et al., 2013; Martin, Meylan, et al., 2015).

Interestingly, UV chroma in males showed a decelerating increase with age after the age of 2–3 years (see Figure 2b) despite a significant increase with body size (see Supporting Information Figure S1). This indicates that the expression of structural UV coloration might eventually drop in the oldest, but not the bigger, males. However, the effect size of this pattern was small, suggesting weak negative effects of senescence. This was expected as UV chroma is under sexual selection, and as such might be maintained later in life to maximize reproductive output. Mechanisms underlying plastic changes late in life in UV chroma are poorly known but may involve environmental conditions during molt such as limited food availability and/or poor thermal conditions (Bajer, Molnar, Török, & Herczeg, 2012; Martin, Le Galliard, et al., 2015). Altogether, the development of sexual dichromatism in UV chroma is in line with our expectation of UV colors being a male sexual signal.

For violet–blue reflectance, an inverse measure of the skin carotenoid content in the common lizard (San‐Jose et al., 2013), sexual dichromatism was more apparent on the chest–belly region where carotenoid content of the skin of males increased faster with age than in females. This result was in line with our hypothesis of carotenoid coloration being involved primarily in sexual selection, with strong carotenoid colors being a male signal. Indeed, sexual dichromatism in yellow–red carotenoid belly coloration in common lizard is important for sex recognition, influences mating success in males, and is a strategic signal of male–male competition among male color morphs (Fitze et al., 2009; Sinervo et al., 2007).

The ontogenetic trajectory of the strength of violet–blue reflectance on the chest–belly region was best explained by a decelerating decrease, and markedly so in males, showing no sign of late‐life decay. This suggests that skin carotenoid content increases throughout life, especially in males, and that it is not subjected to senescence. Given that strong carotenoid coloration is a male signal, and if carotenoids were costly to produce and maintain, we would have expected female carotenoid coloration to be senescent. Hence, our result are in line with the idea that carotenoid coloration can serve as an inexpensive sexual signal (Evans & Sheldon, 2013).

For skin background reflectance, an inverse measure of melanophore concentration and of the density and spacing of iridophores (Grether et al., 2004; San‐Jose et al., 2013), sexual dichromatism arose from a faster increase with age, but overall lower levels, of skin background reflectance in males. This pattern was unexpected given that skin background reflectance is not thought to be under sexual selection in lizards and should hence not show sexual dichromatism (San‐Jose et al., 2013). As skin melanisation tends to increase with age especially in males, an explanation to this pattern could be that color pigments overlay iridophore layers, hence, decreasing the skin background reflectance (Grether et al., 2004), which would be consistent with the positive correlation between these two components. Alternatively, it is possible that changes in skin background reflectance coincide with changes in iridophore layer reflective properties and are linked to sex‐based differences in skin ultra‐structure. This last explanation would further be consistent with the idea that skin background reflectance enhances the chromatic discrimination and the conspicuousness of the yellow–red carotenoid ventral coloration (San‐Jose et al., 2013) and may therefore facilitate the visual assessment of chromatic differences among individual males after sexual maturation. Thus, it remains unclear whether dichromatism in skin background reflectance is a consequence of sexual selection affecting the reflective properties of the iridophore layer, or if it is simply a by‐product of differences in carotenoid content between males and females. Future studies should examine specifically the role of skin background reflectance, and therefore iridophore reflective properties, in sexual selection in the common lizard (Vercken et al., 2007).

Skin background reflectance did not show signs of late‐life decay so that our study supports the view that it might not be subjected to senescence. This is further in line with studies suggesting that structural color components are not particularly costly to produce and maintain and should thus not be expected to drop in later stages of life (San‐Jose et al., 2013).

Finally, we also found consistent variation in the color components across body regions. Indeed, distinct development patterns in UV and pigmentary colors between males and females led to a less marked sexual dichromatism on the throat than on the ventral region in line with the idea that the display of sexual dichromatism is costly in this species. This also implies that the conspicuousness of color traits may influence the balance between sexual and natural selection, and in this case, conspicuousness might enhance natural selection. Furthermore, the different chromaticity of the throat and chest–belly regions may also imply that these regions convey different information regarding intrasexual and intersexual communication (e.g. LeBas & Marshall, 2000), or that one body region serves to enhance visual detection of the other (Grether et al., 2004). Altogether, all color components developed sexual dichromatism, which was stronger on the more concealed ventral region. Our study thus confirms the view that conspicuousness may be a driving force in the diversification of color signals, highlighting the need for further investigation on the link between color conspicuousness and function.

### 4.2 Senescence of body color components

In general, the maintenance and display of a conspicuous structural and/or pigmentary coloration may be costly, and late‐life decrease in the expression of color ornaments is therefore expected from natural selection (Evans et al., 2011). For example, the foot coloration of blue‐footed booby declines with age because older birds are more sensitive to the production and maintenance costs of coloration (Torres & Velando, 2007). The yellow‐red coloration in the common lizard is condition‐dependent and is sensitive to the availability and quality of food and environmental conditions, implying that the maintenance of bright and conspicuous coloration may be costly (Cote, Arnoux, Sorci, Gaillard, & Faivre, 2010; Fitze et al., 2009; San‐Jose, Granado‐Lorencio, & Fitze, 2012; San‐Jose et al., 2013). At the same time, it is also predicted that sexual selection should favor a progressive increase in ornament expression with age to improve mating success (Evans et al., 2011), as found in some studies of late‐life ornamentation (e.g. Galván & Møller, 2009; Grether, Hudon, & Endler, 2001; Weiss, Kennedy, & Bernhard, 2009). Here, despite a longitudinal study from multiple individuals covering a developmental period from sexual maturation to old age where reproductive senescence has been evidenced in the field (Massot et al., 2011), we found no consistent evidence for any of the color components examined to be subjected to senescence, except for ambiguous signs of late‐life decrease in throat UV reflectance in males. Ontogenetic patterns were best described by linear or decelerating growth curves instead of bell‐shaped curves suggesting that, at best, the development rise of body coloration slows down in adults. Thus, our study confirms earlier results in birds where late‐life decline in color ornaments is rarely observed and coloration seems to be proof against senescence (Evans & Sheldon, 2013).

### 4.3 Interactions between color components

The longitudinal study further indicated that consistent interindividual differences existed for all color traits: individuals with higher scores relative to the mean ontogenetic curve maintained on average similarly higher scores throughout their life (i.e. repeatability scores averaging 25%–35%). This suggests that a significant proportion of the color differences expressed during life was determined by genetic or early environmental conditions, as found in other studies highlighting the importance of both genetic and environmental contribution to the maintenance of polychromatism in lizards (e.g. in the common lizard: Sinervo et al., 2007; Vercken et al., 2007; Fitze et al., 2009; Cote et al., 2010). In addition, the individual‐level analysis uncovered that our measure of the skin carotenoid concentration (i.e. a low violet–blue reflectance) was negatively correlated with UV chroma and skin background reflectance. The first correlation could be due to the high absorbance of carotenoids at low spectral wavelength (i.e. below 400–500 nm, Martin et al., 2013), whereas the second suggests that the skin background coloration produced by iridophores in the range 575–700 nm, as defined by San‐Jose et al. (2013), actually extends to the range of absorption of carotenoids. Yet, it is important to notice that some of the differences in mean coloration observed between sexes or between body regions were relatively free from these functional correlations at the individual level. For example, despite negative correlations between structural and pigmentary colorations at the individual level, we found both a higher UV and pigmentary coloration in males relative to females on the CBR. This highlights a potential decoupling between intra‐ and interindividual constraints on body color variation. In other words, structural correlations between color components seem to be overruled by functional correlations, which to our knowledge is a matter that has not been directly researched as of now.

## 5 CONCLUSION

To conclude, we found in this study that UV, pigmentary, and structural color components developed sexual dichromatism thus implying that they may be predominantly under sexual selection. In addition, we found no consistent evidence for color senescence, which support the idea from other studies that sexual ornaments rarely senesce. Differences in the ontogeny of sexual dichromatism between the throat and belly suggest that the function of a given color component can vary with the conspicuousness of the display. Overall, our study supports the view that skin coloration is a composite trait, which conveys multiple signals depending on sex, age, and body location.

## AUTHORS’ CONTRIBUTION

Willem Bonnaffé: statistical analysis, interpretation of the results, writing of the paper. Melissa Martin: data collection, review, and edition of the manuscript prior to submission. Marianne Mugabo: data collection, review, and edition of the manuscript prior to submission. Sandrine Melan: data collection, supervision of the project, review, and edition of the manuscript prior to submission. Jean‐Francois Le Galliard: data collection, supervision of the project, advisor during the writing process, contributed to the writing of the paper, review, and edition of the manuscript prior to submission.

## DATA ACCESSIBILITY

The dataset used for the statistical analysis, featuring UV, pigmentary, and structural color components from 155 male and female individual lizards, measured at least three times throughout their lifespan and across three body regions, will be made available for access in Dryad.
