# Supplementary material for: Ontogenetic trajectories of body coloration reveal its function as a multicomponent nonsenescent signal
Source: Ecol Evol. 2018 Dec 7;8(24):12299–307. doi: 10.1002/ece3.4369 (PMC6308879; doi:10.1002/ece3.4369)
Supplement: Supplementary file 1 [file ECE3-8-12299-s001.pdf]

Spectrophotometric measurements

Spectral properties of the skin of lizards were measured in three areas: the throat, chest, and belly. Measurements were performed using a spectrophotometer (USB2000; Ocean Optics Inc.) calibrated between 200 and 850 nm, a Xenon light source (PX-2) covering 220-750 nm range, and a 400 μm fibre optic probe (R400-7-UV/VIS; Ocean Optics Inc.). Only the 300-700nm range was considered for analysis. Measurements were obtained by placing and bevelling the probe at a 45° angle on the lizards' skin, avoiding black spots, resulting in a reading spot of approximately 1mm^2. Reflectance was measured relatively to a dark and a white diffuse reference background (WS-1; Ocean Optics Inc.). Two measurements were made on each body area of each lizard (for further details see Martin et al. 2013). This allowed us to estimate the repeatability of spectrophotometric measurements by calculating the correlation coefficient between the first and second measurement ($n = 10447$). Overall, we found high repeatability of our measurements ($\rho_{UVchroma} = 0.95$, $\rho_{Rvioletblue} = 0.93$, $\rho_{Yellowchroma} = 0.95$, $\rho_{Rbackground} = 0.87$, $\rho_{dS} = 0.89$).

References

Martin, Mélissa et al. (2013). "Ultraviolet and carotenoid-based coloration in the viviparous lizard Zootoca vivipara (Squamata: Lacertidae) in relation to age, sex, and morphology". In: *Biological Journal of the Linnean Society*. ISSN: 00244066. DOI: 10.1111/bij.12104.

Nakagawa, S and H Schielzeth (2013). "A general and simple method for obtaining R2 from generalized linear mixed-effects models". In: *Methods in Ecology and Evolution* 4.2, pp. 133–142. ISSN: 2041210X. DOI: DOI10.1111/j.2041-210x.2012.00261.x. arXiv: 2746. URL: {\textless}GotoISI{\textgreater}://WOS:000314974800004.

Figure S1: Change in colour with body size.

Change in UV chroma (a, b), violet-blue reflectance (c, d), and background reflectance (e, f) with body size predicted by models (Table S4) for females (a, c, e) and males (b, d, f). Models featured $n_{ind} = 155$ individuals for $n_{obs} = 1156$ observations. The solid lines correspond to changes predicted from best models in Table S4 for each body region (light grey: throat, dark grey: CBR) and sex. Please note differences in y- and x-axis between males and females.

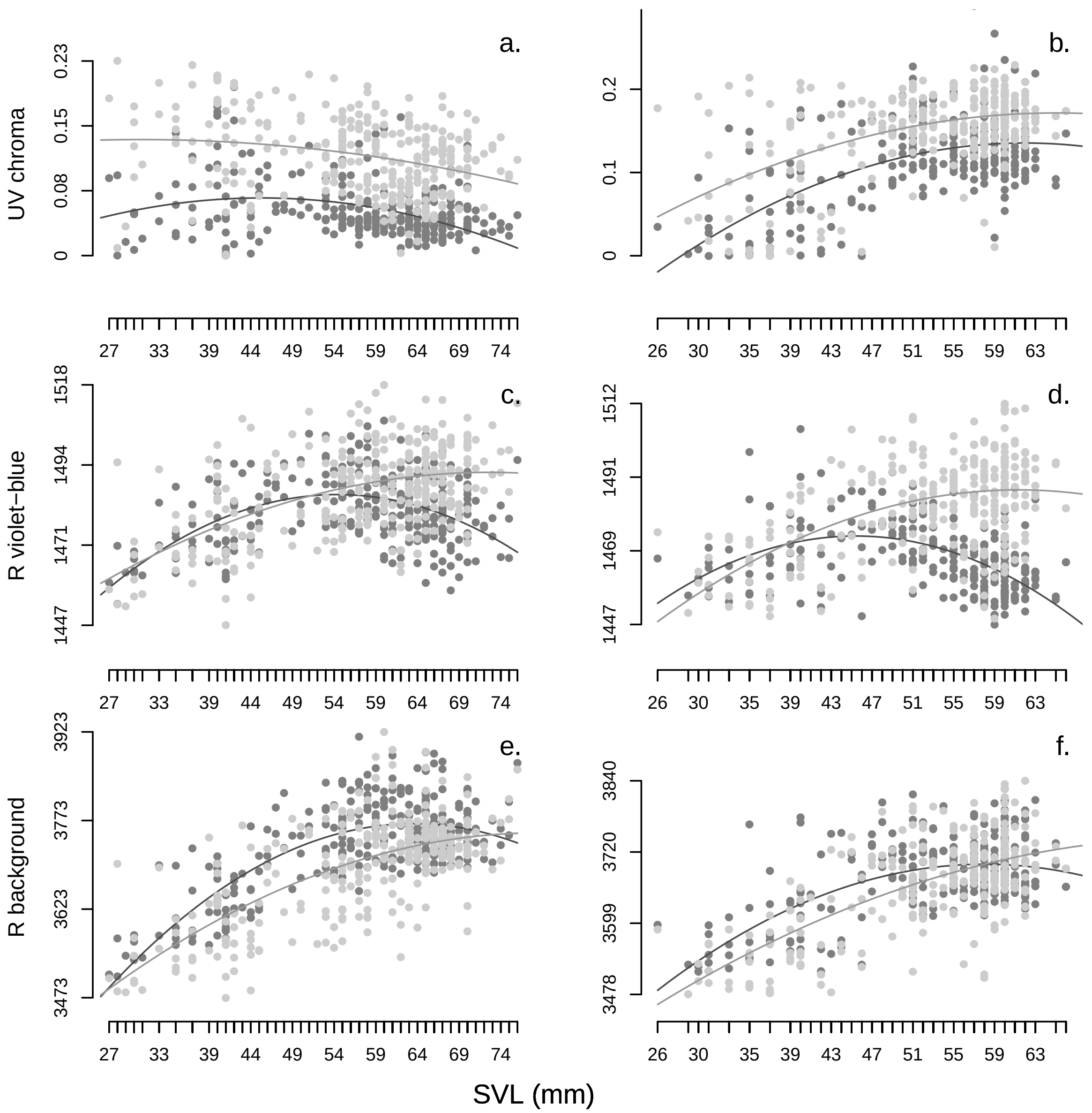

Table S1: Model simplification for the effect of age.
Summary of the step wise model simplification process. The models featured $n_{ind} = 155$ individuals for $n_{obs} = 1156$ observations. The name of the response variable is given along with the deleted terms, the resulting AIC and change in AIC (ΔAIC), likelihood ratio and p-value. Factors are presented in the order of deletion.

Variable	Factor	Step	AIC	ΔAIC	L. ratio	p-value
**UV chroma**	sex:region:age	1	2462.06	-2	0	0.94
	region:age	2	2460.07	-1.75	0.25	0.61
	sex:region:age^2	3	2458.32	-1.02	0.98	0.32
	region:age^2	4	2457.3	0.63	2.63	0.1
**R background**	sex:region:age	1	2907.72	0.57	2.57	0.109
	sex:region:age^2	2	2908.29	-0.41	1.59	0.21
	sex:age^2	3	2907.87	-1.78	0.22	0.6354
	region:age^2	4	2906.1	-1.32	0.68	0.41
**dS**	sex:region:age^2	1	2448.35	-1.67	0.33	0.57
	region:age^2	2	2446.68	0.4	2.4	0.12

Table S2: Estimates of models for the effect of age.
Table summarizing the parameter values for the minimum adequate models describing changes in colour with age. The models featured $n_{ind} = 155$ individuals for $n_{obs} = 1156$ observations. This table displays the name of the variables, the name of the factors, estimated effects, standard error, as well as the degrees of freedom (DF), t-value and p-value of the t tests. Model properties, such as the inter-individual variance (σ_U^2), intra-individual variance (σ^2), AIC, conditional r-squared (i.e. proportion of total variance accounted by fixed and random variance components, library MuMin, R v 3.0.1, Nakagawa and Schielzeth 2013), and intraclass correlation coefficient ($\rho = \frac{\sigma_U^2}{\sigma_U^2+\sigma^2}$), are also given. Note that effects are estimated for standardized variables.

Variable & model properties	Factor	Est. effect	Std. Error	DF	t-value	p-value
**UV chroma**	Intercept	-1.08	0.06	995	-18.24	<0.01
	sex(M)	1.34	0.08	153	15.73	<0.01
$\sigma_U^2 = 0.364$	region(throat)	1.15	0.05	995	21.61	<0.01
$\sigma^2 = 0.641$	age	-0.18	0.04	995	-4.85	<0.01
AIC = 2457.93	age^2	0.07	0.02	995	3.05	<0.01
$r^2 = 0.52$	sex(M):region(throat)	-0.5	0.08	995	-6.62	<0.01
$\rho = 0.362$	sex(M):age	0.61	0.05	995	11.55	<0.01
	sex(M):age^2	-0.23	0.03	995	-7.75	<0.01
**R violet-blue**	Intercept	0.16	0.06	991	2.55	0.01
	sex(M)	-1.12	0.09	153	-12.18	<0.01
$\sigma_U^2 = 0.356$	region(throat)	0.52	0.07	991	7.27	<0.01
$\sigma^2 = 0.682$	age	-0.06	0.05	991	-1.2	0.23
AIC = 2592.27	age^2	-0.07	0.03	991	-2.1	0.04
$r^2 = 0.46$	sex(M):region(throat)	0.85	0.1	991	8.56	<0.01
$\rho = 0.343$	sex(M):age	-0.3	0.08	991	-3.81	<0.01
	sex(M):age^2	0.1	0.04	991	2.36	0.02
	region(throat):age	0.48	0.07	991	6.48	<0.01
	region(throat):age^2	-0.07	0.04	991	-1.56	0.12
	sex(M):region(throat):age	0.52	0.11	991	4.8	<0.01
	sex(M):region(throat):age^2	-0.18	0.06	991	-3.03	<0.01
**R background**	Intercept	0.74	0.06	995	12.31	<0.01
	sex(M)	-0.72	0.08	153	-8.69	<0.01
$\sigma_U^2 = 0.288$	region(throat)	-0.47	0.07	995	-7.06	<0.01
$\sigma^2 = 0.806$	age	0.36	0.05	995	7.63	<0.01
AIC = 2904.77	age^2	-0.23	0.02	995	-12.85	<0.01
$r^2 = 0.29$	sex(M):region(throat)	0.34	0.1	995	3.6	<0.01
$\rho = 0.263$	sex(M):age	0.12	0.05	995	2.35	0.02
	region(throat):age	0.22	0.05	995	4.62	<0.01
**Contrast**	Intercept	0.66	0.06	993	11.13	<0.01
	sex(M)	0.03	0.08	153	0.32	0.75
$\sigma_U^2 = 0.364$	region(throat)	-1.21	0.05	993	-22.87	<0.01
$\sigma^2 = 0.636$	age	0.15	0.05	993	3.4	<0.01
AIC = 2447.08	age^2	-0.04	0.02	993	-1.92	0.05
$r^2 = 0.52$	sex(M):region(throat)	-0.23	0.08	993	-3.05	<0.01
$\rho = 0.364$	sex(M):age	-0.12	0.06	993	-1.82	0.07
	sex(M):age^2	0.1	0.03	993	3.44	<0.01
	region(throat):age	-0.08	0.06	993	-1.45	0.15
	sex(M):region(throat):age	-0.21	0.08	993	-2.76	<0.01

Table S3: Model simplification for the effect of size.
Summary of the step wise model simplification process for models describing changes in colour along body size. The models featured $n_{ind} = 155$ individuals for $n_{obs} = 1156$ observations. The name of the response variable is given along with the deleted terms, the resulting AIC and change in AIC (ΔAIC), likelihood ratio and p-value. Factors are presented in the order of deletion.

Variable	Factor	Step	AIC	ΔAIC	L. ratio	p-value
**UV chroma**	sex:region:SVL^2	1	2268.91	-0.68	1.32	0.25
	sex:region:SVL	2	2268.23	-1.66	0.34	0.56
	region:SVL	3	2266.58	-1.84	0.16	0.68
**R violet-blue**	sex:region:SVL^2	1	2401.32	-1.9	0.1	0.75
**R background**	sex:region:SVL^2	1	2436.81	-1.73	0.27	0.6
	sex:SVL^2	2	2435.08	0.34	2.34	0.13

Table S4: Estimates of models for the effect of size.
Table summarizing the parameter values for the minimum adequate models describing changes in colour along body size. The models featured $n_{ind} = 155$ individuals for $n_{obs} = 1156$ observations. This table displays the name of the variables, the name of the factors, estimated effects, standard error, as well as the degrees of freedom (DF), t-value and p-value of the t tests. Model properties, such as the inter-individual variance (σ^2_U), intra-individual variance (σ^2), AIC, conditional r-squared (i.e. proportion of total variance accounted by fixed and random variance components, library MuMin, R v 3.0.1, Nakagawa and Schielzeth 2013), and intraclass correlation coefficient ($\rho = \frac{\sigma^2_U}{\sigma^2_U+\sigma^2}$), are also given. Note that effects are estimated for standardized variables.

Variable & model properties	Factor	Value	Std. Error	DF	t-value	p-value
**UV chroma**	Intercept	-0.82	0.06	994	-12.92	$<$0.01
	sex(M)	1.17	0.09	153	13.46	$<$0.01
$\sigma^2_U = 0.391$	region(throat)	1.06	0.06	994	18.74	$<$0.01
$\sigma^2 = 0.577$	SVL	-0.23	0.03	994	-8.08	$<$0.01
AIC = 2258.49	SVL^2	-0.12	0.03	994	-4.69	$<$0.01
$r^2 = 0.59$	sex(M):region(throat)	-0.48	0.07	994	-6.87	$<$0.01
$\rho = 0.404$	sex(M):SVL	0.52	0.07	994	7.69	$<$0.01
	sex(M):SVL^2	-0.1	0.04	994	-2.45	0.01
	region(throat):SVL^2	0.07	0.02	994	2.8	0.01
**R violet-blue**	Intercept	0.44	0.07	992	6.63	$<$0.01
	sex(M)	-1.23	0.09	153	-13.97	$<$0.01
$\sigma^2_U = 0.363$	region(throat)	0.15	0.07	992	2.07	0.04
$\sigma^2 = 0.622$	SVL	-0.05	0.04	992	-1.16	0.25
AIC = 2400.01	SVL^2	-0.27	0.03	992	-9.38	$<$0.01
$r^2 = 0.54$	sex(M):region(throat)	1.11	0.08	992	13.72	$<$0.01
$\rho = 0.369$	sex(M):SVL	-0.72	0.08	992	-8.67	$<$0.01
	sex(M):SVL^2	-0.12	0.04	992	-2.7	0.01
	region(throat):SVL	0.43	0.05	992	8.35	$<$0.01
	region(throat):SVL^2	0.16	0.04	992	4.53	$<$0.01
	sex(M):region(throat):SVL	0.61	0.08	992	7.34	$<$0.01
**R background**	Intercept	0.74	0.06	993	12.46	$<$0.01
	sex(M)	-0.7	0.07	153	-9.59	$<$0.01
$\sigma^2_U = 0.236$	region(throat)	-0.69	0.07	993	-9.25	$<$0.01
$\sigma^2 = 0.657$	SVL	0.44	0.04	993	10.91	$<$0.01
AIC = 2436.21	SVL^2	-0.27	0.03	993	-9.86	$<$0.01
$r^2 = 0.53$	sex(M):region(throat)	0.55	0.09	993	6.5	$<$0.01
$\rho = 0.264$	sex(M):SVL	-0.29	0.06	993	-4.52	$<$0.01
	region(throat):SVL	0.15	0.05	993	2.84	$<$0.01
	region(throat):SVL^2	0.14	0.04	993	3.79	$<$0.01
	sex(M):region(throat):SVL	0.31	0.09	993	3.55	$<$0.01
